# Preventive cardiology in pediatrics. A fellow's voice

**DOI:** 10.1016/j.ajpc.2023.100582

**Published:** 2023-09-12

**Authors:** Hannah Hollon

**Affiliations:** Department of Pediatric Cardiology, Children's Hospital Colorado, Denver, CO, United States

**Keywords:** Pediatrics, Overweight, Obesity, Cardiovascular risk

I have always wanted to specialize in preventive health as it is so important to prevent disease, particularly as a pediatrician. However, my fascination with medicine began with the heart. In medical school, I learned that each congenital heart disease is like an intricate puzzle, that when uniquely put together, results in improved blood flow and a chance at a better quality of life. Ever since, I have wanted to become a pediatric cardiologist. However, obesity and weight-related comorbidities are some of the most pressing medical problems facing children today, which is why I chose to spend an extra year of training specializing in pediatric obesity medicine before starting pediatric cardiology fellowship. Through this training, I became interested in combining my two interests into ultimately practicing preventive cardiology after training. I feel that I can make a big impact in children's lives by helping them prevent and treat heart disease so that they can grow into healthy adults. The following piece highlights the unique aspects of preventive cardiology in children.

Atherosclerotic cardiovascular disease and subsequent cardiovascular events are increasingly common and can begin in childhood. Data from the Pathobiological Determinants of Atherosclerosis in Youth (PDAY) study autopsied adolescents and young adults that died of accidental causes and found that 10–30 % of 15–19-year-olds had fatty streaks in the aorta and 2–5 % had fatty streaks in the coronary arteries [1]. This study established that atherogenesis cannot be considered an adult disease alone and identified childhood cardiovascular risk factors as youth smoking, obesity, elevated blood pressure, hyperglycemia, and abnormal lipid levels [1]. Unfortunately, the prevalence of these risk factors is increasing in the setting of the obesogenic environment, as the rates of obesity in childhood have tripled in the last 30 years [2]. Prevention or management of risk factors in childhood can improve cardiovascular outcomes in adulthood.

Prevention of cardiovascular and cardiometabolic disease starts early in childhood, indeed in utero, with optimized nutrition. Heart healthy dietary recommendations are focused on monounsaturated and polyunsaturated fat consumption, limited to 30 % of daily calories [3]. The optimal diet for cardiovascular health should be high in whole, plant-based foods with high fiber content and limited sugar-sweetened beverages. Sedentary time should be minimized, with intermittent active breaks, allowing time for at least 1 h of cumulative physical activity daily [3]. Lastly, children should refrain from smoking or vaping tobacco products and seek cessation counseling if they are currently smoking [3]. While some genetic and epigenetic risk factors cannot be modified, if children maintain these healthy habits throughout childhood and adolescence, their risk of developing elevated body mass index (BMI), blood pressure, glucose, and lipid levels and subsequent early cardiovascular disease is lowered. These strategies are also the basis for first-line treatment should cardiovascular risk factors develop.

Obesity, particularly central obesity that reflects insulin resistance and cardiometabolic risk, is one of the strongest predictors of atherosclerosis [4,5]. There is no one treatment strategy for obesity, but in accordance with United States Preventive Services Taskforce guidelines and the European Prevention Society, the American Academy of Pediatrics (AAP) now recommends providing 26 lifestyle and behavioral treatment hours over 3–12 months in children 6 years old and greater with overweight or obesity [6], [7], [8]. Clinicians can consider weight loss pharmacotherapy in youth 12 years old and up with class I obesity (BMI ≥ 95th percentile) and at least one weight-related comorbidity or class II severe obesity (BMI ≥ 120 % of the 95th percentile). US Food and Drug Administration (FDA)-approved pharmacotherapy options for youth 12 years and older include liraglutide, semaglutide, phentermine, phentermine/topiramate, and orlistat, which lead to a 4.7–16.7 % decrease in BMI depending on the pharmacotherapeutic agent [9], [10], [11]. Metabolic surgery can also be considered in patients with class II severe obesity and at least one weight-related comorbidity and class III severe obesity [12]. Bariatric surgery is the most effective weight loss tool for severe obesity, which has been shown to reduce BMI by 26 % at 5-year post-op follow-up with high rates of diabetes and hypertension remission [13].

Hypertension impacts 3.5 % of children and adolescents and is associated with increased carotid atherosclerosis, left ventricular hypertrophy, and all-cause mortality [14,15]. Blood pressure is measured at every well child visit beginning at age 3. Definitions of elevated blood pressure by age group are presented in Table 1 [15]. Further guidance on diagnosing hypertension in children and adolescents can be found in the AAP Clinical Practice Guideline for Hypertension [15]. The most common etiology of elevated blood pressure in pediatrics is excess weight, presumed in association with insulin resistance. The mainstay of treatment is insulin-sensitizing lifestyle change with the goal of reducing blood pressure to less than the 90th percentile or 130/80 mmHg. Pharmacotherapy is reserved for patients who fail a trial of lifestyle intervention or those with severe hypertension and end-organ damage. This approach reduces end-organ damage and prevents progression of hypertension into adulthood, which further increases risk of cardiovascular disease [16,17].

**Table 1:** Definitions of hypertension by age group.

Age Range	< 13 years old	≥ 13yo
Elevated Blood Pressure	≥ 90th percentile to < 95th percentile	120/79–129/79 mm Hg
Stage I Hypertension	≥ 95th percentile to < 95th percentile + 12 mm Hg	130/80–139/89 mm Hg
Stage II Hypertension	≥ 95th percentile + 12 mm Hg	≥ 140/90 mm Hg

Prediabetes is present in one in five adolescents with even greater prevalence in adolescents with obesity [18]. Prediabetes is defined with a hemoglobin A1c between 5.7 % and 6.4 %, but hyperglycemia is forestalled by hyperinsulinism in youth with resilient pancreatic function. Elevations in triglycerides and/or diminished high density lipoprotein (HDL)-cholesterol may also help identify insulin resistance [19]. Early identification of prediabetes allows for prompt lifestyle intervention and prevention of type 2 diabetes. Again, the mainstay of treatment is lifestyle change. There is no standard of care for pharmacotherapy to treat prediabetes. Clinicians can consider metformin up to 2000 mg/day in patients 10 years and older, particularly in patients with a family history of type 2 diabetes and acanthosis on physical exam. If weight loss is a desired treatment strategy for prediabetes, clinicians can also consider glucagon-like peptide-1 agonists described above for children 12 years and older for improvement in weight and insulin sensitivity. Evaluation and management of type 2 diabetes in youth is beyond the scope of this review.

Dyslipidemia is present in one fifth of all children and adolescents [20] and up to 43 % of youth with obesity [21]. Lipid screening recommendations are controversial in pediatrics, but the AAP recommends universal fasting lipid screening at 9–11 years old and again at 17–21 years old [6]. This can also help identify those with familial hypercholesterolemia (FH). Diagnostic cut offs for dyslipidemia are as follows: total cholesterol ≥ 200 mg/dL, HDL ≤ 40 mg/dL, LDL ≥ 130 mg/dL, and triglycerides ≥ 100 mg/dL in children less than 10 years old or triglycerides ≥ 130 mg/dL in children over 10 years of age [14]. Many dyslipidemias can be managed with dietary and physical activity interventions per the risk reduction strategies described above. Lipid-lowering medications, including statin therapy, can be considered in patients ages 10 years and older after 6 months of lifestyle therapy based on the risk stratification provided in Table 2 [3]. Elevated triglycerides in the range of 200–499 mg/dL are managed with lifestyle therapy alone, and triglyceride levels ≥ 500 mg/dL can be treated with fibrates, niacin, or fish oil to prevent pancreatitis [3].

**Table 2:** Dyslipidemia risk stratification (from 2011 NHLBI/AAP guidelines).

High-Level Risk Factors	Moderate-Level Risk Factors	No Risk Factors
- Positive family history of early atherosclerotic cardiovascular disease - BMI at the ≥ 97th percentile - Hypertension on pharmacotherapy - Current tobacco exposure - Presence of high-risk conditions - Diabetes, Type 1 or Type 2 - Chronic kidney disease - Kawasaki disease with coronary aneurysm - Post-orthotic heart transplant	- BMI ≥ 95th, < 97th percentile - Hypertension not on pharmacotherapy - HDL-cholesterol ≤ 40 mg/dL - Presence of moderate-risk conditions - Kawasaki disease with resolved coronary aneurysm - Chronic inflammatory disease (SLE, JRA, HIV) - Nephrotic syndrome	
LDL ≥ 130 mg/dL	LDL ≥ 160 mg/dL	LDL ≥ 190 mg/dL

With growing evidence supporting atherosclerosis development in childhood in high-risk patients, there needs to be greater emphasis on prevention beginning early, even in the womb with interventions to improve maternal diet and lifestyle habits. As healthcare professionals, our goal is to prevent the diagnosis and management of the risk factors and chronic diseases detailed in this review. This starts with fostering healthy behavioral habits related to nutrition, physical activity, sedentary time, sleep, and mental health. These habits have the potential to make a substantial impact on a patient's life when started in childhood, leading to accumulated protective factors against atherosclerotic cardiovascular disease in adulthood.

Special acknowledgements to: Anurag Mehta, MD^1^, Michele Mietus-Snyder, MD^2^

^1^ Virginia Commonwealth University, Department of Cardiology, Richmond, VA; ^2^ Children's National Hospital, Department of Pediatric Cardiology, Washington, D.C.

## Declaration of Competing Interest

The authors declare that they have no known competing financial interests or personal relationships that could have appeared to influence the work reported in this paper.
